# Cytomegalovirus seropositivity is associated with glucose regulation in the oldest old. Results from the Leiden 85-plus Study

**DOI:** 10.1186/1742-4933-9-18

**Published:** 2012-08-28

**Authors:** Sijia Chen, Anton JM de Craen, Yotam Raz, Evelyna Derhovanessian, Ann Vossen CTM, Rudi Westendorp GJ, Graham Pawelec, Andrea B Maier

**Affiliations:** 1 Department of Gerontology and Geriatrics, Leiden University Medical Center, Albinusdreef 2, Leiden, 2333 ZA, The Netherlands; 2 Netherlands Consortium for Healthy Ageing, Leiden University Medical Center, Leiden, the Netherlands; 3 Tübingen Ageing and Tumour Immunology Group, Center for Medical Research, University of Tübingen Medical School, Tübingen, Germany; 4 Department of Medical Microbiology, Leiden University Medical Center, Leiden, the Netherlands

**Keywords:** Cytomegalovirus, Seropositivity, IgG antibody level, Type 2 diabetes, HbA1c, Non-fasting glucose, C-reactive protein, Elderly, Oldest olds

## Abstract

**Background:**

Cytomegalovirus (CMV) infection has been reported to contribute to the pathogenesis of type 1 diabetes and post-transplantation diabetes. However, CMV infection has not been evaluated as a possible risk factor for type 2 diabetes. Our aim was to investigate potential associations between CMV seropositivity, CMV IgG antibody level and glucose regulation in the oldest old.

**Results:**

CMV seropositive subjects were more likely to have type 2 diabetes (17.2% vs 7.9%, p = 0.016), had a higher level of HbA1c (p = 0.014) and higher non-fasting glucose (p = 0.024) in the oldest olds. These associations remained significant after adjustment for possible confounders. CMV IgG antibody level was not significantly associated with glucose regulation (all p > 0.05).

**Conclusions:**

In the oldest old, CMV seropositivity is significantly associated with various indicators of glucose regulation. This finding suggests that CMV infection might be a risk factor for the development of type 2 diabetes in the elderly.

## Background

The increasing prevalence of type 2 diabetes can be attributed to the growing proportion of elderly and the higher incidence of type 2 diabetes among younger individuals in developed countries. Obesity, inactivity and ageing are associated with insulin resistance. When pancreatic islets progressively fail to enhance production of insulin as compensation for insulin resistance, insulin deficiency, chronic hyperglycaemia and eventually type 2 diabetes will develop. However, while only one third of individuals with insulin resistance will ultimately develop type 2 diabetes, many others do not because their β-cells are able to respond adequately to the increased demand for insulin [1,2]. The reason for this heterogeneity is not completely understood. In the pathogenesis of type 2 diabetes, several genetic [3], epigenetic [4] and environmental [5] factors are believed to contribute, while chronic inflammation is also suggested to be a characteristic feature of the disease. Systemic markers in type 2 diabetes are shifted towards a more pro-inflammatory status, including elevated C-reactive protein (CRP) and fibrinogen levels and greater numbers and a more activated state of various leukocyte populations [2,6]. Despite accumulating evidence that chronic inflammation is a risk factor for type 2 diabetes [2,6,7], there is only limited knowledge whether infection by specific pathogens contributes to inflammation and the subsequent incidence of type 2 diabetes.

A chronic stressor for the immune system is the common herpes virus cytomegalovirus (CMV) which establishes persistent, life-long infections and can become reactivated periodically [8,9]. CMV seropositivity was clustered as one parameter of the “Immune Risk Profile” [10] in the very elderly and has been linked to diseases and syndromes with an inflammatory component including cancer [11,12], cardiovascular disease [13], frailty [14], functional impairment [15] and cognitive impairment [16,17], and mortality [18]. Therefore, active CMV infection or reactivation from a latent state is considered potentially a cofactor for inflammatory disease [12]. Several findings suggest that persistent CMV infection may participate in the pathogenesis of type 2 diabetes. CMV may accelerate immunosenescence by inducing the accumulation of late-differentiated CD4+ and CD8+ T-cells which produce pro-inflammatory cytokines and thereby generate a more pro-inflammatory environment [19-21]. Furthermore, pro-inflammatory cytokines were reported to have deleterious effects on pancreatic β-cells which could lead to an insufficient response to insulin resistance, resulting in onset of type 2 diabetes [22]. Moreover, non-symptomatic CMV infection is known to be a risk factor for the development of new-onset post-transplantation diabetes (PTDM) [23]. In one study, CMV RNA was identified in the pancreas of patients with type 2 diabetes, localized primarily in the islets of Langerhans [24], but a different study failed to detect CMV DNA in the pancreas of such patients [25]. However, recently it was shown that human pancreatic β-cells are susceptible to CMV infection [26]. At the population level, a clear positive association between CMV infection and type 2 diabetes has not yet been reported [27-30].

The aim of the present study was to explore the association between CMV seropositivity, CMV IgG antibody level, and indicators of glucose regulation assessed by means of the frequency of diagnosis of type 2 diabetes, level of glycated haemoglobin (HbA1c) and level of non-fasting glucose in a cohort of the oldest old in the general population.

## Results

Table 1 summarizes the baseline characteristics of the study population stratified by CMV serostatus (n = 549). All participants were 85 years of age, the majority were CMV-seropositive (n = 435, 79.2%). CMV-seropositive participants were more often female, had lower education level and lower income (all p < 0.05).

**Table 1:** Characteristics of the participants, aged 85 years (n = 549), stratified by CMV serostatus

	**CMV-serostatus**	
	**positive (n = 435)**	**negative (n = 114)**
Gender: women, n (%)	303 (69.7)	65 (57.0)
Education: low, n (%)	313 (72.5)	45 (39.5)
Income: low, n (%)	238 (55.5)	41 (36.6)
BMI (kg/m^2^), mean (SD)	27.4 (4.5)	26.6 (4.6)
Smoking: ever, n (%)	199 (46.1)	62 (54.4)
Alcohol (units/week), median (IQR)	0 (0-3)	1 (0-7)
Comorbidities:		
Diabetes, n (%)	75 (17.2)	9 (7.9)
COPD, n (%)	51 (12.0)	12 (10.9)
Parkinson, n (%)	11 (2.6)	1 (0.9)
Cancer, n (%)	68 (16.0)	27 (24.5)
Arthritis, n (%)	140 (33.0)	31 (28.2)
Sumscore of cardiovascular comorbidities, mean (SD)	3.9 (19.7)	3.0 (13.3)
Total number of comorbidities, mean (SD)	4.8 (19.8)	3.8 (13.4)
Total number of medications, mean (SD)	3.3 (2.7)	3.0 (2.6)
CRP (mg/L)*, mean (SD)	0.8 (1.7)	1.0 (1.8)

*CRP (mg/L) is logtransformed.

BMI: body mass index. CRP: C-reactive protein. COPD: chronic obstructive pulmonary disease. Sumscore of cardiovascular comorbidities: hypertension, myocardial infarction, angina pectoris, claudicatio intermittens, arterial surgery and stroke. Total number of comorbidities: sumscore of cardiovascular comorbidities, COPD, Parkinson, Cancer, Arthritis.

**Figure 1:**
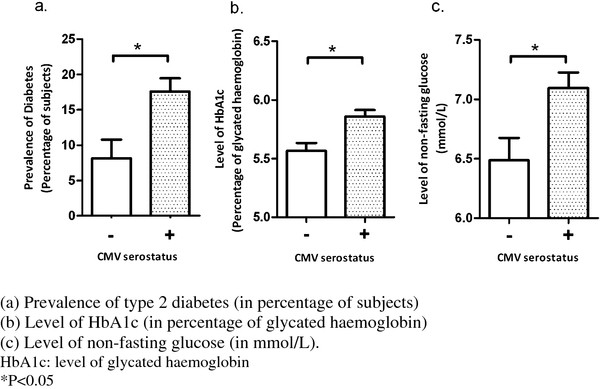
**Comparison of glucose regulation in relation to CMV serostatus. **( **a**) Prevalence of type 2 diabetes (in percentage of subjects), ( **b**) Level of HbA1c (in percentage of glycated haemoglobin). ( **c**) Level of non-fasting glucose (in mmol/L). HbA1c: level of glycated haemoglobin *P < 0.05.

Table 2 shows associations between CMV seropositivity and indicators of glucose regulation expressed as the frequency of diagnosis of type 2 diabetes, level of HbA1c and level of non-fasting glucose (n = 547). The frequency of type 2 diabetes was 15% (n = 84) of the entire population. Diagnosis of type 2 diabetes (p = 0.014), level of HbA1c (p = 0.016) and non-fasting glucose (p = 0.024) were all significantly higher in CMV seropositive participants. These associations remained significant after adjustment for possible confounders. The findings are depicted in Figure 1.

**Table 2:** Associations between CMV serostatus and indicators of glucose regulation

		**OR (95% CI)**	**P value**
Diagnosis of type 2 diabetes (0 = no, 1 = yes)	Model 1	2.44 (1.18;5.05)	0.016
	Model 2	2.35 (1.04;5.31)	0.041
	Model 3	2.42 (1.07;5.49)	0.034
HbA1c (%)	Model 1	1.37 (1.07;1.77)	0.014
	Model 2	1.41 (1.05;1.90)	0.024
	Model 3	1.44 (1.07;1.95)	0.017
Non-fasting glucose* (mmol/l)	Model 1	2.23 (1.11;4.48)	0.024
	Model 2	2.46 (1.05;5.76)	0.039
	Model 3	2.50 (1.06;5.88)	0.036

*Non-fasting glucose (mmol/l) is logtransformed.

OR: Odds Ratio, 95% CI: Confidence Interval.

Model 1 (n = 547): Not adjusted (crude model).

Model 2 (n = 510): Adjusted for gender, income, education, smoking, body mass index, total number of comorbidities and total number of medications.

Model 3 (n = 510): Model 2 and adjusted for logtransformed C-reactive protein.

Table 3 shows the association between CMV IgG antibody level and the diagnosis of type 2 diabetes, level of HbA1c and non-fasting glucose, in CMV seropositive participants (n = 432). CMV IgG antibody level was not associated with any of the markers of glucose regulation (all p > 0.05).

**Table 3:** Associations between CMV IgG antibody level and indicators of glucose regulation

		**Beta (SE)**	**P value**
Diagnosis of type 2 diabetes (0 = no, 1 = yes)	Model 1	1.44 (1.17)	0.220
	Model 2	1.70 (1.27)	0.182
	Model 3	1.86 (1.27)	0.144
Hba1c(%)	Model 1	0.47 (0.39)	0.226
	Model 2	0.49 (0.42)	0.240
	Model 3	0.56 (0.42)	0.181
Non-fasting glucose* (mmol/l)	Model 1	1.19 (1.40)	0.185
	Model 2	2.38 (1.52)	0.119
	Model 3	2.44 (1.52)	0.110

*Non-fasting glucose (mmol/l) is logtransformed.

SE: Standard error.

Model 1 (n = 432): Not adjusted (crude model).

Model 2 (n = 404): Adjusted for gender, income, education, smoking, body mass index, total number of comorbidities and total number of medications.

Model 3 (n = 404): Model 2 and adjusted for logtransformed CRP.

## Discussion

In the present study, CMV seropositivity was associated with indicators of glucose regulation in terms of more frequent diagnoses of type 2 diabetes, elevated levels of HbA1c and non-fasting glucose in very elderly people infected with this virus. The titer of CMV IgG antibody, however, was not significantly associated with these indicators. Our findings suggest a role for CMV infection in the pathogenesis of type 2 diabetes in the elderly.

CMV might be involved in accelerating pancreatic failure to compensate for insulin resistance via at least two possible mechanisms. First, it could influence the pancreatic cells directly; secondly, it might act indirectly by influencing the immune system which in turn affects the pancreas. Consistent with the first possibility is the report that CMV may infect and reside in pancreatic cells without causing cytopathic effects but nonetheless influencing insulin production directly after repeated reactivations [24]. Additionally, infection of human pancreatic β-cells with CMV induced the release of pro-inflammatory cytokines and increased cellular immunogenicity [26]. The indirect effects of CMV could be exerted via infected monocyte production of IL-1β which induces TNF-α production in human pancreatic duct cells, driving cells into apoptosis and thus compromising β-cell function [24,31]. Other components of the immune system, influenced by prolonged CMV infection, could hypothetically also contribute to a more pro-inflammatory environment, which is an important feature of type 2 diabetes [2]. CMV seropositivity is associated with accumulations of potentially senescent late- differentiated T-cells and elevated numbers of CD4+ and CD8+ effector cells [32] which are more likely to produce pro-inflammatory cytokines [21].

Thus far, no relationships between glucose regulation and CMV seropositivity had been confirmed at the population level [27-30]. Three earlier studies investigated notably younger participants with comorbidities in relatively small cohorts [27-29]. Age, ethnicity, income and education (socioeconomic factors) have not always been considered important confounders for CMV and diseases, although this is very likely to be the case especially for the latter [33,34]. In 1000 participants aged 45–84 years in the Multiethnic study of atherosclerosis (MESA), associations between CMV infection, pathogen burden > 3 and diagnoses of type 2 diabetes were indeed observed. After adjustment for demographic covariates (race/ethnicity and education), however, all associations became non-significant, suggesting no aetiological role for pathogens/CMV in the occurrence of type 2 diabetes [29].

Three possible explanations for why positive relationship of CMV seropositivity with glucose regulation emerges in the oldest old can be given. First, the direct deleterious effects of CMV infection on pancreatic cells might only become significant after a long period of activation and reactivation of CMV and therefore only detectable in the oldest old. Second, systemic inflammation may interfere with the action of insulin on cells by suppressing intracellular insulin signal transduction [35]. This possible indirect systemic effect of CMV infection on the immune system would also take time to develop. Third, our study population of 85-year-olds is of course highly selected for longer-than-average survival, and has thus already lost individuals with other known risk factors for cardiovascular diseases and type 2 diabetes. These risk factors could have overshadowed the impact of CMV infection in glucose regulation in younger people.

An alternative explanation posits that hyperglycaemia may impair host defenses, predispose to infection and therefore leads to a higher seroprevalence of CMV in diabetic patients [36]. Thus, higher prevalence of CMV would be a result, not a cause, of disease. However, CMV infection is often acquired during childhood. Once the host is infected, the virus persists in various tissues. The chance of becoming infected with CMV is highly influenced by socioeconomic conditions and close contacts allowing the virus to spread [33,34]. It is therefore unlikely that diabetes precedes CMV infection, but it still cannot be ruled out that there are undetected predisposing factors to both diabetes and CMV infection.

In the present study, CMV IgG antibody titer did not associate significantly with diagnoses of type 2 diabetes in the oldest old. Although CMV antibody level has been reported to associate with mortality among community-dwelling older adults [37,38], it is still unclear what the clinical implications of CMV antibody level on the onset of diseases may be and what the effect of chronological age on CMV antibody level is [39,40]. Recently, CMV antibody level has been reported not to be related to any major genetic determinants either [41].

The cross-sectional nature of the relationship between CMV seropositivity and indicators of glucose regulation is one limitation of our study. One cross-sectional study is not sufficient to prove whether the contribution of CMV infection is causative. This relationship should be confirmed in studies with a prospective design in younger participants with long-term follow-up and regular monitoring of incidence of CMV infection. A second limitation is that the blood samples were collected under non-fasting conditions. It is therefore likely that we have underestimated the effect of CMV seropositivity on glucose level, which would increase the robustness of our findings. Still, the old age of the participants could limit the applicability of these findings to the general population, but the fact that our study population is relatively large and all participants were 85 years of age could also be seen as a strength. It might be feasible to study the effect of CMV seropositivity only in such special participants as the impact of CMV may become pronounced after years of latent infection plus periodic reactivations. The large size of our study and the homogeneity of the age could have made the impact of CMV easier to determine.

Our finding that CMV seropositivity is associated with more diagnoses of type 2 diabetes in the oldest olds leads to the hypothesis that CMV seropositivity may facilitate the onset of type 2 diabetes in the long term. It is crucial to demonstrate the association using a prospective study design, starting with a younger study population, as CMV infection may be a “hidden” cause of morbidity [18].

## Conclusions

The present study is the first to show that CMV seropositivity is positively independently associated with indicators of glucose regulation. Our findings shed light on the pathogenesis of type 2 diabetes in the very elderly. More research needs to be performed to determine causality in the relationship of CMV infection with type 2 diabetes.

## Methods

### Participants’ characteristics

The Leiden 85-plus Study is a prospective population-based follow-up study of inhabitants of the city of Leiden, the Netherlands. The over-all aim of this study was to investigate determinants of successful ageing. All inhabitants who reached the age of 85 years were eligible to participate. There were no selection criteria on health, functioning or demographic characteristics. Enrolment took place between 1997 and 1999. In total, 599 individuals participated in the study, 87% of all eligible individuals. Details were provided in a previous publication [42]. All participants gave informed consent. For persons with severe cognitive impairment, informed consent was given by a guardian.

### CMV serostatus and CMV IgG antibody level

For the current analysis, we included 549 participants with information on CMV serostatus and antibody level determined by the CMV IgG kit (ETI-CYTOK-G PLUS DiaSorin, Saluggia, Italy) based on enzyme immunoassay technology. Anti-CMV IgG antibody levels were expressed as antibody units/ml.

### Diagnosis of type 2 diabetes, level of HbA1c and determination of non-fasting glucose

Diagnosis of type 2 diabetes at baseline was defined by: 1) reporting the use of oral anti-diabetes medication or the use of insulin based on information obtained from the participant’s pharmacist; 2) treatment by diet, or registration by a general practitioner as having diabetes; or 3) non-fasting glucose of ≥ 11.0 mmol/l. Blood samples were collected in the early morning. HbA1c and glucose in plasma were determined using fully automated analysers (Hitachi 747 and 911; Hitachi, Ltd, Tokyo, Japan). HbA1c results were reported in DCCT units as percentage of haemoglobin, HbA1c can also be expressed in mmol/mol, according to the IFCC reference method. DCCT units can be converted into IFCC units using the following equation: IFCC-HbA1c (mmol/mol) = [DCCT-HbA1c (%) - 2.15] x 10.929.

### Characteristics of participants and possible confounders

At baseline, all participants were visited by a research nurse at their place of residence for interviews and performance tests: information on income and education was obtained; the measurements for body mass index (BMI) were done for all participants. Education levels were dichotomized into high and low. The category “high” consisted of participants who had more years of education than elementary school only. Elementary school or less years of education were considered as “low”. Scales of income were dichotomised into high and low. Smoking history was dichotomized in never and ever. The category “ever” consisted of participants who were current or past smokers. For alcohol drinking habits, units of alcohol per week were listed. For each participant, the total number of comorbidities was calculated using medical histories obtained from the general practitioner or treating nurses and included cardiovascular comorbidities, chronic obstructive pulmonary disease (COPD), Parkinson, cancer, arthritis.

The sumscore of cardiovascular comorbidities consisted of hypertension, myocardial infarction, angina pectoris, claudicatio intermittens, arterial surgery and stroke. In addition, information on the use of medication was obtained from the participant’s pharmacist. Blood samples were collected early in the morning under non-fasting conditions. Plasma levels of C-reactive protein (CRP) were measured with the use of a fully automated Hitachi 911 device.

### Statistical analysis

The associations between CMV infection (expressed as CMV seropositivity and as CMV IgG antibody level) and indicators of glucose regulation (diagnosis of type 2 diabetes, level of HbA1c and non-fasting glucose level) were assessed using logistic regression analysis and linear regression analysis with adjustments in three models. First, analysis was not adjusted for possible confounders (crude model). Second, the analysis was adjusted for gender and factors known to be associated with CMV infection and glucose regulation (income, education, smoking, BMI, total number of comorbidities (sumscore of cardiovascular comorbidities, COPD, Parkinson, cancer, arthritis) and total number of medications as an estimate of the severity of the comorbidities). Third, CRP was included to further elucidate the effect of systemic inflammation on the relationship between CMV infection and glucose regulation. Distributions of continuous variables were examined for normality and logarithmically transformed where appropriate (for CRP and non-fasting glucose). The statistical Package for the Social Sciences (SPSS) program for windows, version 17 was used for data analysis. Figures were drawn using Graph Pad Prism version 5. P-values < 0.05 were considered statistically significant.

## Competing interests

The authors declare that they have no competing interests.

## Authors’ contributions

SC, YR carried out the data analysis and drafted the manuscript. ED, GP, RGHW, AJMC ABM, participated in design and the coordination of the study and helped draft the manuscript. All authors read and approved the final version of the manuscript.

## Funding

This work was supported by unrestricted grants from the Netherlands Organisation of Scientific Research (ZonMw), the Ministry of Health, Welfare, and Sports and the Netherlands Genomics Initiative/Netherlands Organization for scientific research (NGI/NWO; 05040202 and 050-060-810 NCHA), and the EU-funded Network of Excellence Lifespan (FP6 036894), as well as by DFG Pa 361/14-1 (to GP).
